# In vitro apical extrusion of debris and instrumentation time following root canal instrumentation with Reciproc and Reciproc Blue instruments and a novel stainless steel rotary system (Gentlefile) versus manual instrumentation

**DOI:** 10.34172/joddd.2023.39271

**Published:** 2023-11-11

**Authors:** Ahmad Nouroloyouni, Shahriar Shahi, Amin Salem Milani, Sara Noorolouny, Robab Farhang, Aysan Yousefi Azar

**Affiliations:** ^1^Department of Endodontics, Faculty of Dentistry, Ardabil University of Medical Science, Ardabil, Iran; ^2^Department of Endodontics, Faculty of Dentistry, Tabriz University of Medical Science, Tabriz, Iran; ^3^Department of Pediatric Dentistry, Faculty of Dentistry, Ardabil University of Medical Science, Ardabil, Iran

**Keywords:** Root canal preparation, Root canal therapy, Tooth apex

## Abstract

**Background.:**

This study compared apical extrusion of debris and instrumentation time following root canal instrumentation with Reciproc, Reciproc Blue, and Gentlefile (GF) rotary instruments versus the manual step-back technique.

**Methods.:**

This in vitro study was conducted on 80 extracted mandibular premolars with mature apices and a root curvature of<10°. The teeth were randomly assigned to 4 groups (n=20), standardized regarding working length, and placed in pre-weighed vials. The root canals were instrumented with Reciproc, Reciproc Blue, and GF systems and the manual step-back technique in the four groups. The vials containing the collected debris were then dried and weighed. The instrumentation time was also recorded for each group. Data were analyzed with one-way ANOVA and post hoc Games-Howell test (α=0.05).

**Results.:**

Minimum apical debris extrusion was noted in Reciproc, followed by Reciproc Blue, GF, and manual technique (*P*<0.05). Pairwise comparisons showed significantly lower apical extrusion of debris in the Reciproc compared with GF (*P*=0.015) and manual instrumentation (*P*=0.011) groups. The Reciproc system also had the shortest instrumentation time, followed by Reciproc Blue, GF, and manual instrumentation (*P*<0.05). Pairwise comparisons showed significant differences between all the systems (*P*<0.05) except between Reciproc and Reciproc Blue (*P*>0.05) in this respect.

**Conclusion.:**

Although all systems caused apical extrusion of debris, manual instrumentation caused maximum extrusion of debris. In contrast, the Reciproc system was superior to others regarding minimal apical extrusion of debris and the shortest instrumentation time.

## Introduction

 Root canal instrumentation is an important step in endodontic treatment, which can be performed by manual or rotary instruments.^1^ Manual instrumentation is time-consuming; also, using hand files in narrow and curved canals may cause procedural errors such as ledge formation or canal transportation due to the low flexibility of hand files. Using nickel-titanium files with higher flexibility can decrease procedural errors in narrow and curved canals.^2^

 Apical extrusion of debris commonly occurs during the chemomechanical preparation of the root canal system, irrespective of the instrumentation technique, which can cause postoperative pain and edema. Despite precise control of the working length, apical extrusion of pulpal residues, debris, necrotic tissues, microorganisms, and irrigants into the periapical region is common in endodontic treatment, leading to flare-ups.^3-5^

 Although all instrumentation techniques cause apical extrusion of debris, the amount of extruded debris varies in different systems and techniques.^6-10^ It has been reported that lower extrusion of debris is probably associated with a better treatment outcome.^11^

 Reciproc (VDW, Munich, Germany) is a single-file system built of martensitic NiTi wire and is used in a reciprocating motion. It has two cutting edges and three file sizes with an “S” cross-section: R25 (0.25/.08), R40 (0.40/.06), and R50 (0.50/.05). Reciproc Blue (VDW, Munich, Germany) is a modernized version with the same cross-sectional shape and geometry, but a new NiTi alloy was introduced in 2016.^12,13^

 The Gentlefile system (GF, Tornado; MedicNRG, Kibbutz Afikim, Israel) is a novel approach to endodontic procedures using stainless steel.^14^ This system’s unique design incorporates specialized files with a multipart structure. In the apical third of the file, there is a central braided cable less than 0.15 mm in width. This cable has a second smaller wire, measuring < 0.20 mm, wrapped around it. Moving towards the middle and coronal regions of the instrument, a third wire, no greater than 0.35 mm, coils over the second. The last 0.5 mm of the apical end is sharpened at a 45° angle to create a passive, non-cutting tip. The files have a consistent 4% taper and an inactive, passive tip. Notably, the tip diameters of 0.21, 0.23, 0.26, 0.29, and 0.34 mm deviate from the standard ISO dimensions for endodontic instruments.^14^

 The manufacturer claims that the GF has been designed to minimize pressure and maximize cleaning efficiency. The unique design of this product is intended to minimize the unnecessary removal of tooth structure while still allowing for effective cleaning of the root canal system and preservation of the original root canal anatomy. The GF files are composed of stainless steel and exhibit exceptional flexibility due to their distinctive design, rendering them remarkably resistant to fractures.^14^

 The GF instrument demonstrated reduced apical transportation within the 5–7-mm segment, in contrast to ProTaper Next. Additionally, the GF instrument surpassed other techniques in efficiently removing the smear layer. The coarse external texture of GF files facilitates the even expulsion of debris from the root canal, ensuring consistent and uniform shaping of the root canal walls.^15^

 The GF files rotate at a speed of 6500 rpm and have a nearly negligible torque due to their specific material composition and design. This characteristic effectively safeguards against root canal deformation. Additionally, this system features a specific portable handpiece with adjustable angulation, providing the files with high flexibility. This system has six files of different colors and sizes: gray (coronal file, 20 mm, 022 tip), black (25 mm, 034 tip), green (25 mm, 029 tip), blue (25 mm, 026 tip), red (25 mm, 023 tip), and yellow (25 mm, 021 tip).^14,15^

 A recent randomized controlled single-masked clinical trial also suggested that longer instrumentation times could lead to increased damage to the tissues surrounding the tooth apex. This could increase postoperative pain. Therefore, assessing the duration of instrumentation appears beneficial for reducing postoperative discomfort.^16^

 Considering the differences in the design of different root canal instrumentation systems, variations in the amount of apically extruded debris and instrumentation time are expected. Accordingly, this study compared the amount of apically extruded debris and preparation time following root canal instrumentation with Reciproc, Reciproc Blue, and GF systems compared with the manual step-back technique. The null hypothesis was that no significant difference would be found in the amount of apically extruded debris and instrumentation time between the four groups.

## Methods

 The present in vitro study was carried out on 80 mandibular premolars extracted as part of orthodontic treatment and unrelated to this study (ethical approval code: IR.ARUMS.REC.1399.362). The sample size was calculated at n = 20 in each group according to a previous study by Nevares et al,^17^ with a study power of 80%, α = 0.05, and β = 0.2 using PASS 11.

 The collected teeth had almost straight roots and underwent periapical radiography from the mesiodistal and buccolingual aspects to assess the root canal morphology. Teeth with more than one root canal or one apical foramen and those with immature apex, root curvature > 10°, previous endodontic treatment, root resorption, or calcification were excluded.

 To eliminate the organic debris from the root surfaces, the teeth were immersed in 5.25% sodium hypochlorite. Calculus was also removed by a hand scaler. The teeth were then stored in 0.5% chloramine T solution until use.

 Access cavities were prepared using a round Endo Z bur (Dentsply Maillefer, Tulsa, Oklahoma, USA) and contra-angle handpiece at high speed under water coolant. Working length was determined using a #10 K-file (Mani Inc., Tochigi, Japan). The file was introduced into the canal until its tip was visible at the apex; 1 mm was subtracted from this length to determine the working length. Teeth with a primary file size of #15-20 were selected.

 Eppendorf tubes were first weighed with a digital scale (CPA225D, Sartorius, Germany) with a precision of 0.01 mg. The method introduced by Myers and Montgomery^18^ was adopted to quantify the amount of extruded debris. For this purpose, the teeth were placed in Eppendorf tubes through a stopper. The end of each Eppendorf tube was cut, and the tube was placed in a larger vial. Cyanoacrylate glue was used to fill the gaps around the stopper to prevent the leakage of irrigants into the vial. A smaller vial was also placed in the larger vial such that the root end was placed in the smaller vial. The small vials were coded and weighed three times by an endodontist before use by a digital scale with 0.01 mg accuracy. A 27-gauge needle was used to balance the air pressure inside and outside the vial. A rubber dam was also placed on the tooth to ensure masked conduction of the procedure.^19^ The teeth were then randomly divided into four groups (n = 20) as follows:

- Group 1: Root canal instrumentation was performed using stainless steel hand K-files with a length of 21 mm and a taper of 0.02 (Mani, Tohnichi, Japan) with a quarter-pull motion and the step-back technique. The apical part was prepared up to the #25 K-file.
- Group 2: Reciproc R25 single file rotary system
- Group 3: Reciproc Blue R25 single file rotary system
- Group 4: The GF rotary system with a 4% taper was used to prepare the apical region. The preparation was performed in an orderly manner using the following tips: #gray 022 tip, #black 034 tip, #green 029 tip, #blue 026 tip, and #red 023 tip. The instruments were used with their respective handpieces operating at 6500 rpm. A picking motion with direct apical pressure was applied for 5 seconds.

A second endodontist instrumented the root canals in each group according to the manufacturers’ instructions. Root canals that were accidentally over-instrumented were excluded and replaced. Root canal irrigation was performed by a side-vented syringe. Rotary instruments were discarded after preparing three root canals. Irrigation was performed with 8 mL of distilled water during the preparation of each root canal. The irrigation needle was used 3 mm shorter than the working length.

 After completing root canal instrumentation, the root canals were rinsed with 2 mL of distilled water and dried with paper points. The teeth were then removed from Eppendorf tubes. To collect the apically extruded debris adhering to the roots, the apical part of the roots was also rinsed with 2 mL of distilled water. All the tubes were then incubated at 37 °C for 15 days before weighing for the irrigant to evaporate. After incubation, the tubes were checked with the naked eye to ensure complete evaporation of the irrigant and were then weighed three times by the same operator of primary weighing. The mean of the three measurements was calculated and recorded. The primary weight of the tubes was subtracted from the final weight to quantify the amount of apically extruded debris. The instrumentation time was also recorded for each file system from the time of introduction of the file into the canal until the completion of instrumentation.

 Data were analyzed by one-way ANOVA followed by the post hoc Games-Howell test using SPSS 23 at an 0.05 level of significance.

## Results

###  Apical extrusion of debris

Table 1 presents the mean amount of extruded debris in the four groups. As shown, minimum apical extrusion of debris was noted in Reciproc, followed by Reciproc Blue, GF, and manual technique. Considering the normal distribution of data (*P* > 0.05), ANOVA was applied to compare the four groups regarding apical extrusion of debris, which revealed a significant difference in this regard between the four groups (*P* = 0.001).

**Table 1:** Mean amount of extruded debris (mg) in the four groups (n = 20)

**Groups**	**Mean**	**SD**	**Minimum**	**Maximum**	***P***** value**
Gentlefile	2.383	1.87	0.16	7.20	0.130
Manual instrumentation	2.677	2.19	0.14	7.26	0.176
Reciproc	0.9195	0.621	0.17	2.31	0.200
Reciproc Blue	1.389	0.731	0.13	2.78	0.200

 Considering the non-homogeneity of variances according to Levene’s test (*P* = 0.000), pairwise comparisons of the groups were carried out using the Games-Howell test (Table 2). The Reciproc group showed significantly lower apical extrusion of debris compared with the GF (*P* = 0.015) and manual instrumentation (*P* = 0.011) groups. No other significant differences were noted (*P* > 0.05).

**Table 2:** Pairwise comparisons of the groups regarding the apical extrusion of debris using the Games-Howell test

**Group (I)**	**Group (J)**	**Mean difference (I-J)**	**Std. error**	***P***** value**
Gentlefile	Manual	0.294-	0.644	0.968
	Reciproc	1.46	0.442	0.015*
	Reciproc Blue	0.994	0.450	0.149
Manual	Reciproc	1.76	0.508	0.011*
	Reciproc Blue	1.29	0.516	0.087
Reciproc	Reciproc Blue	0.469-	0.214	0.145

*Statistically significant.

###  Instrumentation time 

Table 3 presents the instrumentation times in the four groups. The Reciproc system had the shortest instrumentation time, followed by Reciproc Blue, GF, and manual instrumentation. Considering the normal distribution of data (*P* > 0.05), ANOVA was applied to compare the instrumentation time between the four groups, which revealed a significant difference (*P* = 0.000). Considering the non-homogeneity of variances according to Levene’s test (*P* = 0.006), pairwise comparisons of the groups were carried out using the Games-Howell test (Table 4). The results showed significant differences between all the groups in instrumentation time (*P* < 0.05) except for the difference between Reciproc and Reciproc Blue (*P* = 0.423).

**Table 3:** Instrumentation times in the four groups (n = 20)

**Groups**	**Mean**	**SD**	**Minimum**	**Maximum**	***P*** value*
Gentlefile	172.65	37.707	96	234	0.200
Manual instrumentation	239.90	50.719	146	346	0.073
Reciproc	72.20	16.237	41	91	0.200
Reciproc Blue	82.40	24.633	48	138	0.200

*Significance level is 0.05.

**Table 4:** Pairwise comparisons of the groups regarding instrumentation times using the Games-Howell test

**Group (I)**	**Group (J)**	**Mean difference (I-J)**	**Std. error**	***P***** value**
Gentlefile	Manual	-67.250	14.132	0.000*
	Reciproc	100.45	9.180	0.000*
	Reciproc Blue	90.250	10.071	0.000*
Manual	Reciproc	167.700	11.908	0.000*
	Reciproc Blue	157.500	12.608	0.000*
Reciproc	Reciproc Blue	-10.200	6.597	0.423

*Statistically significant.

## Discussion

 This study compared the amount of apically extruded debris and instrumentation times following root canal treatment with Reciproc, Reciproc Blue, and GF systems compared with the manual step-back technique. The null hypothesis was that no significant difference would be found in the amount of apically extruded debris and instrumentation times between the four groups.

 Several factors affect the apical extrusion of debris through the apex. Thus, the effect of confounding factors should be controlled to assess the pure effect of the instrumentation technique on the apical extrusion of debris. The type of tooth is an influential factor in this respect. Most previous studies used single-canal, single-apex teeth with straight roots (6‒10° curvature) to simplify root canal preparation and achieve predictable results.^20,21^ Also, in the clinical setting, most endodontic treatments are performed on teeth with mild to moderate curvature. Thus, single-canal premolars were used in the present study to better simulate the clinical setting. The experience of the clinician is another influential factor in debris extrusion. Thus, one skilled operator experienced in working with all tested instrumentation systems performed all the root canal procedures in this study.^22^ The type of irrigant also affects the apical extrusion of debris.^23^ Parirokh et al^23^ showed that the use of 5.25% sodium hypochlorite caused greater extrusion of debris than 2.5% sodium hypochlorite. The majority of similar previous studies used distilled water as an irrigant.^20,21,24^ Thus, distilled water was used in the present study as well. Using distilled water as an irrigant prevents weight gain of mineral deposits after the evaporation of the irrigant. Also, in the present study, the vials were placed in a desiccator to minimize the possible humidity inside the vials. Moreover, no pressure was applied during irrigant injection into the root canal system to minimize its apical extrusion.

 Finally, the Myers and Montgomery^18^ model was adopted to assess the apical extrusion of debris in this study. However, this model cannot simulate the pressure created by the periapical tissues around the apex, which has been criticized by many studies.^20,21^ In this model, the tooth apex is suspended in the air, and there is no barrier against the extrusion of debris. Some authors have suggested using floral foam around the apex to simulate periapical pressure.^25^ However, such foams absorb the debris and irrigant. Another study used agarose gel to simulate periapical tissues.^26^ However, the homogeneous consistency of the gel is different from the texture of periapical tissues. Thus, a simulation of periapical pressure was not performed in this study. On the other hand, considering the possible effect of apical diameter on the extrusion of debris,^11^ a #25 master apical file was used in all teeth to standardize the apical size.

 The present results showed apical extrusion of debris in all the groups. Minimum apical extrusion of debris was noted in Reciproc, followed by Reciproc Blue, GF, and manual technique (P < 0.05). Pairwise comparisons showed significantly lower apical extrusion of debris in the Reciproc group compared with GF (*P* = 0.015) and manual instrumentation (P = 0.011) groups. Thus, the null hypothesis of the study was rejected. In line with the present results, Buldur et al,^22^ Boijink et al,^7^ Toyoğlu and Altunbaş,^27^ Topçuoğlu et al,^28^ and De-Deus et al^29^ showed significantly higher apical extrusion of debris in manual instrumentation compared with the use of different rotary systems. Higher apical extrusion of debris in manual instrumentation compared with rotary systems is due to the fact that the movement of rotary files guides the debris towards the orifice while apical pressure applied to the file in push-and-pull movements in the step-back technique pushes the debris apically in manual instrumentation.^30^

 As mentioned earlier, the apical extrusion of debris in the Reciproc group was significantly lower than in the manual instrumentation group in the present study. Similarly, De-Deus et al^31^ reported significantly lower apical extrusion of debris with Reciproc compared with manual instrumentation and the ProTaper Universal rotary system. De-Deus et al^29^ showed significantly lower extrusion of debris in Reciproc compared with ProTaper and WaveOne. The minimum apical extrusion of debris in the Reciproc and Reciproc Blue groups in the present study was also consistent with the results of other studies.^32^ Moreover, Doğanay Yıldız and Arsalan^33^ showed lower apical extrusion of debris in Reciproc compared with Reciproc Blue. However, Bürklein and Schäfer^20^ indicated that multi-file rotary systems had lower apical extrusion of debris than single-file rotary systems. Variations in the results can be due to different methodologies, differences in debris collection assemblies, and the use of different types of digital scales and files. To the best of the authors’ knowledge, the present study is the first to compare the apical extrusion of debris following the use of reciprocating files and the Gentlefile multi-file system versus manual instrumentation.

 Higher apical extrusion of debris in the GF group compared with Reciproc may be due to the use of a higher number of files in the GF multi-sequence system, which can increase the production and extrusion of debris due to higher frequency of filing and irrigation. Also, the reciprocating movement of single-file systems has a lower tendency to push the debris apically compared with continuous rotation movement.^21^ Furthermore, the twisted nature of wires in the GF files causes greater and more invasive preparation of the root canal walls compared with single-file systems and results in greater production and extrusion of debris. Moreover, differences in apical debris extrusion can also be attributed to the preparation technique, cross-sectional design of instruments, and their taper. The Reciproc system has an S-shaped cross-section and a non-cutting tip.^34^

 Assessment of instrumentation times in the present study revealed that the Reciproc system had the shortest preparation time, followed by Reciproc Blue, GF, and manual instrumentation (*P* < 0.05). Pairwise comparisons showed significant differences between all the systems (*P* < 0.05) except between Reciproc and Reciproc Blue (P > 0.05). In accordance with the present results, Bürklein and Schäfer^20^ reported a shorter preparation time of mandibular central incisors in the use of Reciproc compared with WaveOne, Mtwo, and ProTaper. Also, Kucukyilmaz et al^35^ demonstrated a shorter preparation time of single-canal canine teeth with Reciproc compared with ProTaper and OneShape. Similarly, Topçuoğlu et al^36^ showed minimum instrumentation time with Reciproc. Bürklein et al^37^ demonstrated that the Reciproc file decreased root canal preparation time by 60% compared with the Mtwo multi-file system. The results of the abovementioned studies were all consistent with the present results. In the current study, the GF system had the maximum instrumentation time among the tested engine drive systems, probably because it is a multi-sequence system, while Reciproc and Reciproc Blue are single-file systems.

 This study had an in vitro design, which limits the generalization of results to the clinical oral environment. For example, the physical pressure caused by periodontal tissue against the extrusion of debris was not simulated in this study. Also, the clinical significance of small variations in the apical extrusion of debris is not yet known. Further investigations are required to assess the effect of the type and load of bacteria adhering to the extruded debris and the host response on the severity of postoperative pain and edema. Also, clinical trials are required on postoperative pain using different single-file rotary systems. Apical extrusion of debris in curved canals should also be investigated in future studies.

## Conclusion

 Although all the systems caused apical extrusion of debris, manual instrumentation caused maximum extrusion of debris. In contrast, the Reciproc system was superior to others regarding minimal debris extrusion and the shortest instrumentation time.

## Acknowledgments

 We would like to express our great appreciation to Dr. Mojdeh Kalantar Motamedi for her valuable help, comments, and translation.

## Competing Interests

 The authors declare that she has no known competing financial interests or personal relationships that could have influenced the work reported in this paper.

## Data Availability Statement

 The data used to support the findings of this study were supplied by the corresponding author under license, and the data will be available upon reasonable request. Requests for access to these data should be made to the corresponding author within 12 months of publication.

## Ethical Approval

 The protocol of this study was approved by the Ethics Committee of Ardabil University of Medical Sciences (Ethical approval code: IR.ARUMS.REC.1399.362).

## References

[1] Peters OA (2004). Current challenges and concepts in the preparation of root canal systems: a review. J Endod.

[2] Calberson FL, Deroose CA, Hommez GM, De Moor RJ (2004). Shaping ability of ProTaper nickel-titanium files in simulated resin root canals. Int Endod J.

[3] Siqueira JF Jr (2003). Microbial causes of endodontic flare-ups. Int Endod J.

[4] Salem Milani A, Froughreyhani M, Taghiloo H, Nouroloyouni A, Asghari Jafarabadi M. The effect of antibiotic use on endodontic post-operative pain and flare-up rate: a systematic review with meta-analysis. Evid Based Dent. 2022. 10.1038/s41432-021-0205-z. 35165442

[5] Nouroloyouni A, Lotfi M, Shahi S, Rahimi S, Noorolouny S, Salem Milani A (2023). Postoperative pain after endodontic treatment of mandibular molars with two different instrumentation techniques: a randomized clinical trial. Dent Res J (Isfahan).

[6] Uslu G, Özyürek T, Yılmaz K, Gündoğar M, Plotino G (2018). Apically extruded debris during root canal instrumentation with Reciproc Blue, HyFlex EDM, and XP-endo Shaper nickel-titanium files. J Endod.

[7] Boijink D, Costa DD, Hoppe CB, Kopper PMP, Grecca FS (2018). Apically extruded debris in curved root canals using the WaveOne Gold reciprocating and Twisted File Adaptive systems. J Endod.

[8] Amaral AP, Limongi P, Fontana CE, de Martin AS, da Silveira Bueno C, Pinheiro SL (2019). Debris apically extruded by two reciprocating systems: a comparative quantitative study. Eur J Dent.

[9] Keskin C, Sivas Yilmaz Ö, Inan U (2020). Apically extruded debris produced during glide path preparation using R-Pilot, WaveOne Gold Glider and ProGlider in curved root canals. Aust Endod J.

[10] Yammine SD, Jabbour EA (2021). Apically extruded debris following programmed over instrumentation of curved canals with three nickel titanium rotary instruments. Eur J Dent.

[11] Tinaz AC, Alacam T, Uzun O, Maden M, Kayaoglu G (2005). The effect of disruption of apical constriction on periapical extrusion. J Endod.

[12] Pedullà E, Grande NM, Plotino G, Gambarini G, Rapisarda E (2013). Influence of continuous or reciprocating motion on cyclic fatigue resistance of 4 different nickel-titanium rotary instruments. J Endod.

[13] Ozlek E, Gunduz H (2021). The effect of heat-treated single-file systems on dentinal crack formation. Niger J Clin Pract.

[14] Moreinos D, Dakar A, Stone NJ, Moshonov J (2016). Evaluation of time to fracture and vertical forces applied by a novel Gentlefile system for root canal preparation in simulated root canals. J Endod.

[15] Htun PH, Ebihara A, Maki K, Kimura S, Nishijo M, Okiji T (2020). Cleaning and shaping ability of Gentlefile, HyFlex EDM, and ProTaper Next instruments: a combined micro-computed tomographic and scanning electron microscopic study. J Endod.

[16] Oliveira PS, Ferreira MC, Paula NGN, Loguercio AD, Grazziotin-Soares R, da Silva GR, et al. Postoperative pain following root canal instrumentation using ProTaper Next or Reciproc in asymptomatic molars: a randomized controlled single-blind clinical trial. J Clin Med 2022;11(13). 10.3390/jcm11133816. PMC9267392 35807101

[17] Nevares G, Xavier F, Gominho L, Cavalcanti F, Cassimiro M, Romeiro K (2015). Apical extrusion of debris produced during continuous rotating and reciprocating motion. ScientificWorldJournal.

[18] Myers GL, Montgomery S (1991). A comparison of weights of debris extruded apically by conventional filing and Canal Master techniques. J Endod.

[19] Koçak S, Koçak MM, Sağlam BC, Türker SA, Sağsen B, Er Ö (2013). Apical extrusion of debris using self-adjusting file, reciprocating single-file, and 2 rotary instrumentation systems. J Endod.

[20] Bürklein S, Schäfer E (2012). Apically extruded debris with reciprocating single-file and full-sequence rotary instrumentation systems. J Endod.

[21] Silva EJ, Carapiá MF, Lopes RM, Belladonna FG, Senna PM, Souza EM (2016). Comparison of apically extruded debris after large apical preparations by full-sequence rotary and single-file reciprocating systems. Int Endod J.

[22] Buldur B, Hascizmeci C, Aksoy S, Nur Aydin M, Guvendi ON (2018). Apical extrusion of debris in primary molar root canals using mechanical and manual systems. Eur J Paediatr Dent.

[23] Parirokh M, Jalali S, Haghdoost AA, Abbott PV (2012). Comparison of the effect of various irrigants on apically extruded debris after root canal preparation. J Endod.

[24] Al-Omari MA, Dummer PM (1995). Canal blockage and debris extrusion with eight preparation techniques. J Endod.

[25] Sharma R, Kumar V, Logani A, Chawla A, Sharma S, Koli B (2020). Effect of gravity on periapical extrusion of irrigating solution with different irrigation protocols in immature anterior teeth. Eur Endod J.

[26] Mitchell RP, Yang SE, Baumgartner JC (2010). Comparison of apical extrusion of NaOCl using the EndoVac or needle irrigation of root canals. J Endod.

[27] Toyoğlu M, Altunbaş D (2017). Influence of different kinematics on apical extrusion of irrigant and debris during canal preparation using K3XF instruments. J Endod.

[28] Topçuoğlu G, Topçuoğlu HS, Akpek F (2016). Evaluation of apically extruded debris during root canal preparation in primary molar teeth using three different rotary systems and hand files. Int J Paediatr Dent.

[29] De-Deus G, Neves A, Silva EJ, Mendonça TA, Lourenço C, Calixto C (2015). Apically extruded dentin debris by reciprocating single-file and multi-file rotary system. Clin Oral Investig.

[30] Ferraz CC, Gomes NV, Gomes BP, Zaia AA, Teixeira FB, Souza-Filho FJ (2001). Apical extrusion of debris and irrigants using two hand and three engine-driven instrumentation techniques. Int Endod J.

[31] De-Deus G, Brandão MC, Barino B, Di Giorgi K, Fidel RA, Luna AS (2010). Assessment of apically extruded debris produced by the single-file ProTaper F2 technique under reciprocating movement. Oral Surg Oral Med Oral Pathol Oral Radiol Endod.

[32] Capar ID, Arslan H, Akcay M, Ertas H (2014). An in vitro comparison of apically extruded debris and instrumentation times with ProTaper Universal, ProTaper Next, Twisted File Adaptive, and HyFlex instruments. J Endod.

[33] Doğanay Yıldız E, Arslan H (2019). The effect of blue thermal treatment on endodontic instruments and apical debris extrusion during retreatment procedures. Int Endod J.

[34] Veltri M, Mollo A, Mantovani L, Pini P, Balleri P, Grandini S (2005). A comparative study of Endoflare-Hero Shaper and Mtwo NiTi instruments in the preparation of curved root canals. Int Endod J.

[35] Kucukyilmaz E, Savas S, Saygili G, Uysal B (2015). Evaluation of apically extruded debris and irrigant produced by different nickel-titanium instrument systems in primary teeth. J Contemp Dent Pract.

[36] Topçuoğlu HS, Demirbuga S, Topçuoğlu G (2020). Evaluation of apically extruded debris during the removal of canal filling material using three different Ni-Ti systems and hand files in teeth with simulated apical root resorption. Int Endod J.

[37] Bürklein S, Hinschitza K, Dammaschke T, Schäfer E (2012). Shaping ability and cleaning effectiveness of two single-file systems in severely curved root canals of extracted teeth: Reciproc and WaveOne versus Mtwo and ProTaper. Int Endod J.

